# Biointegrative Design (BinD): An Interdisciplinary Paradigm for Sustainable Biohybrid Systems

**DOI:** 10.1002/gch2.70162

**Published:** 2026-09-30

**Authors:** Aslan Miriyev

**Affiliations:** ^1^ Physical AI Laboratory Department of Mechanical Engineering Ben‐Gurion University of the Negev Beer‐Sheva Israel

**Keywords:** biohybrid systems, Biointegrative Design, biological dynamics, interdisciplinarity, living machines, physical artificial intelligence, sustainability

## Abstract

The expansion of robotics and automation has been accompanied by increasing accumulations of e‐waste and other persistent materials in natural environments. Biohybrid systems, which combine synthetic components with living matter, offer a fundamentally different path grounded in biodegradability and the zero‐footprint approach. Existing design approaches often produce predefined structures that fail to leverage the dynamic capabilities of living matter. Treating growth, regeneration, and adaptation as intrinsic design variables changes how biohybrid systems are conceived, integrated, and fabricated from the outset. Here, Biointegrative Ddesign (BinD) is proposed as a design paradigm in which biological dynamics are intrinsic variables. Comprising four main stages—bioconceptualization, living subsystem design, supporting subsystem design, and integration and prefabrication—BinD constitutes a continuous, iterative design process. This article shows how BinD crosses disciplinary boundaries to chart a path toward living machines that grow, adapt, and degrade, while transforming fabrication processes, application areas, and business models. It also outlines a universal blueprint for the institutional shifts needed to support BinD and other emerging interdisciplinary paradigms. Together, these contributions lay the groundwork for sustainable engineered systems that align their entire life cycle, from fabrication to degradation, with the natural environment.

## Graphical abstract

Biohybrid systems integrate living and synthetic components, yet no established design methodology can accommodate their biological dynamics. Biointegrative Design (BinD) proposes a paradigm in which growth, regeneration, and adaptation are intrinsic design variables from system conception. Organized into four iterative stages, BinD advances circular engineering across robotics, medicine, and ecological monitoring, while outlining the institutional structures required for its interdisciplinary adoption and its broader implications for fabrication, application areas, and business models.

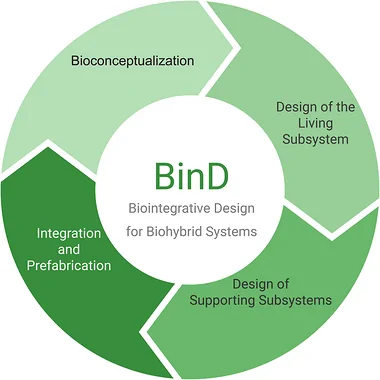

## 1 Background

The continuous advancement and expansion of robotics and automation in recent decades has led to a surge in the number of machines produced and a consequent increase in their ecological footprint. Tremendous amounts of plastic, e‐waste and other persistent materials have already accumulated, causing long‐term contamination of soil and water worldwide. To address this dire global challenge, scientists across disciplines are developing mechanisms with which to contain the ecological impact of synthetic machines, including cost‐efficient recycling technologies and the use of recyclable materials [1]. A more recent, far‐reaching vision proposes that robotics research pursue a zero‐footprint goal by actively integrating biodegradable materials and living cells in robot design and fabrication, so that the resulting systems retain their intended function during operation and leave no persistent environmental footprint after their functional life [2].

Machines that inherently combine synthetic components with living counterparts are biohybrid systems [3]. In the last two decades, biohybrid systems have advanced from the relatively primitive frog muscle explant coupled to a polymeric skeleton that swam under open‐loop electrical stimulation [4] to a diversity of sophisticated examples, including optogenetically controlled self‐healing walking robots [5], autonomously swimming fish powered by mechanoelectrical feedback [6], pumps with flow‐loop biochemical feedback [7], antagonistic muscle pair joint actuators for object manipulation [8], and tissue‐based living diodes capable of directional signal computation [9]. Despite the rapid pace of technical progress in the field, involving vascularization, innervation, and the scalable manufacture of living actuators [10], biohybrid systems lack methodologies for their informed design, a fundamental gap that limits their capabilities. These and other issues were highlighted in a recent field review [3] and are being addressed by a new, dedicated Defense Advanced Research Projects Agency (DARPA) program, HyBRIDS [11].

Conventional engineering design tools were built for synthetic systems with fixed geometries and predetermined material properties. Living matter, by contrast, is inherently dynamic, and conventional design methodologies and the bioinspiration design paradigm [12] lack tools to address these living dynamics. To date, a design methodology that accounts for systems in which growth, adaptation, and degradation are core engineering capabilities does not exist. During the culture of skeletal muscle tissues, for example, the tissues spontaneously contract and remodel, often shrinking away from fixed scaffolds until actuation fails. Indeed, design methods to predict or compensate for this behavior are lacking [8, 10].

Furthermore, biohybrid systems in robotics have been dominated by animal‐derived components, largely due to the direct mapping between their biological functions and engineering requirements, such as muscle tissues used for actuation [13]. A broader biological palette, including plant, fungal, and microbial systems, offers new and, in some cases, fundamentally distinct capabilities, all of which are governed by the laws of living matter that current design frameworks do not accommodate.

A complementary alternative to animal‐derived tissues can be found in plant‐based components with the capacity to self‐assemble structures, harvest energy through photosynthesis, and respond dynamically to environmental cues [14]. The incorporation of living plant tissues as functional components in devices enables biohybrids that grow, self‐repair, and interact with their environment by exploiting plants’ intrinsic physiological processes, such as light harvesting, filtration, or chemical and electrochemical sensing [15, 16]. Similarly, fungal networks contribute structural adaptability, environmental sensing, and robust self‐reinforcing growth. The capacity of the latter to form dynamic, load‐bearing networks offers opportunities for living substrates and adaptive structural materials [17]. Microbial consortia, in turn, confer metabolic and self‐organizing capabilities that support sensing, catalysis, and chemical conversion [18].

Each biohybrid system discussed above requires a dedicated design methodology built around the system's biological dynamics. Such a design methodology will unlock capabilities that synthetic systems cannot replicate and will open pathways to engineering solutions with inherently lower environmental footprints. As a novel scientific frontier, the development and implementation of design methodologies for living machines, which blur the line between biological organisms and engineered machines, will require not only scientific breakthroughs, but also proactive changes at the institutional, ethical, policymaking, and societal levels.

This article proposes the Biointegrative Design (BinD) paradigm as an interdisciplinary methodology for the design of sustainable biohybrid systems, highlights its broader impacts across related domains, and discusses the relevant challenges and implications as the paradigm matures toward becoming a fundamental design methodology for a more sustainable future. In addition, the article presents a universal blueprint with which to scale BinD and other interdisciplinary paradigms by removing institutional silos and supporting the creation of shared cross‐domain languages.

## 2 Biointegrative Design Methodology

Here, the Biointegrative Design (BinD) paradigm, which treats the biological dynamics of living matter as intrinsic engineering variables from the conception of biohybrid design and development, is introduced (Figure 1). While the Physical Artificial Intelligence (PAI) paradigm recently established that the co‐evolution of body, morphology, and control is essential to achieve nature‐like intelligence [19, 20], a rigorous methodology for the physical integration of living, dynamic matter in biohybrid systems is lacking.

**FIGURE 1:**
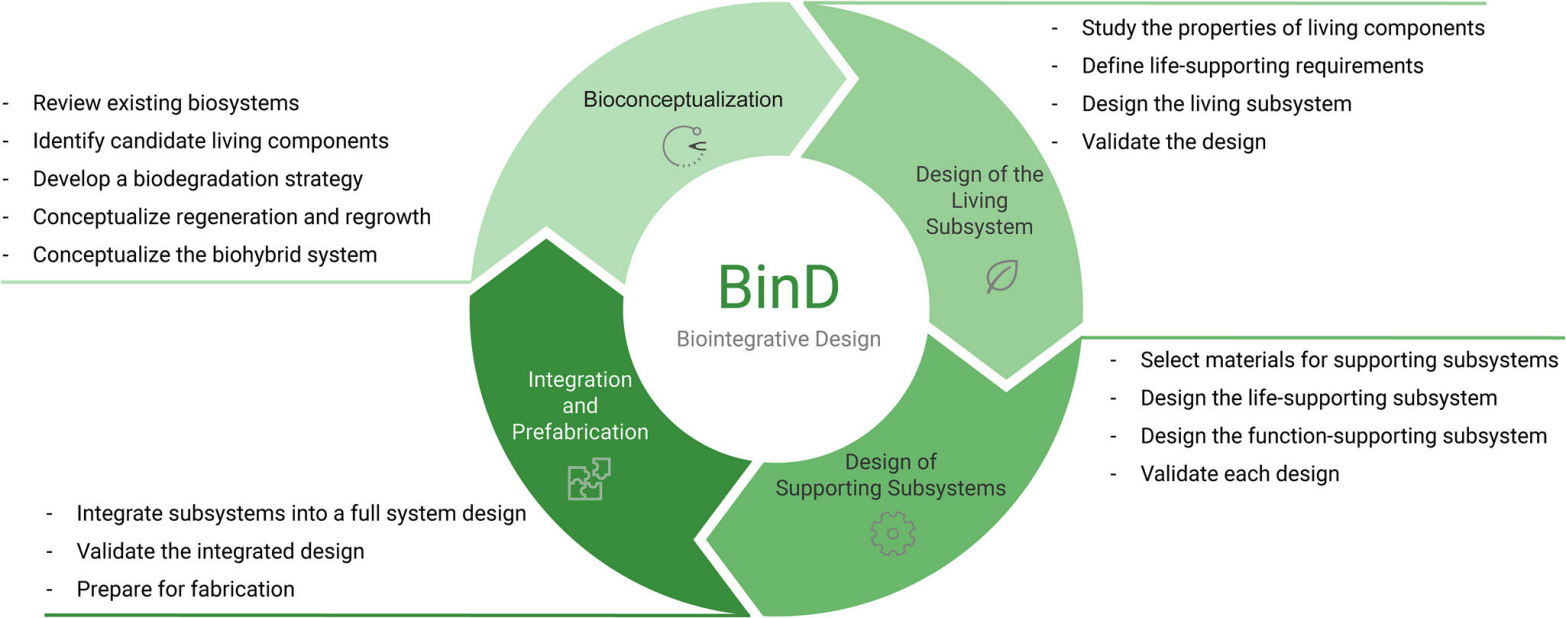
Biointegrative Design (BinD)—a methodology for biohybrid system design in which biological dynamics are intrinsic engineering variables from design conception. BinD organizes the design process into four interrelated stages—bioconceptualization, living subsystem design, supporting subsystem design, and integration and prefabrication—which can progress sequentially or iteratively. All stages may be revisited for optimization as the design evolves. The resulting biohybrid system is prepared for fabrication while remaining amenable to iterative refinement.

The BinD paradigm comprises four main stages that are executed in a continuous, iterative design process. During the first stage, termed bioconceptualization, the overall concept of the biohybrid system is developed. Based on the desired robotic function, this stage defines the “biological operating window” and establishes the thermal, chemical, and nutrient fluxes required for system homeostasis as primary design constraints. At the same stage, the environmental, social, and economic criteria relevant to the intended application and life cycle are also established, with the energy and resources required for manufacturing considered where relevant. The relevant existing biosystems are reviewed, and viable candidates for the living components are identified and selected. Subsequently, the growth dynamics, degradation and regeneration strategies, and interactions of the biosystem candidates with synthetic components are considered to complete system conceptualization.

After establishing the overall concept and its biological operating window, the next stage in BinD, the design of the living subsystem, involves a detailed study of the properties of the selected living component to understand its specific living dynamics and to define its life‐supporting requirements. The living components may include, for instance, tissues, organoids, plant structures, fungal networks, or microbial consortia, each possessing distinct requirements for growth, viability, and integration. This design stage shifts from fixed computer‐aided design (CAD) structures to models that account for nonlinear, multi‐parameter‐driven biological dynamics. Environmental conditions, stimuli, and interface requirements are designed to support the functionality of the biological components. At the end of this and each of the two subsequent stages, the design is validated to ensure that the output of the current and previous stages of the design process is technologically and ethically suitable (as discussed below) for the desired functional profile and consistent with the sustainability criteria established during bioconceptualization.

Once the living subsystem has been defined, the design protocol shifts to the development of the supporting subsystems, which comprise the synthetic components needed to enable and sustain the living subsystem. The BinD paradigm distinguishes between the life‐supporting subsystem, which ensures biological viability, and the function‐supporting subsystem, which enables the target functionality of the biohybrid system. To illustrate this distinction, a nutrient‐providing microfluidic channel setup can be considered a life‐supporting subsystem, while a thin, polymeric layer adjacent to a biological muscle fiber tissue strip, which provides directionality to the fiber contractions, qualifies as a function‐supporting subsystem. Supporting subsystems may also include, among others, structural scaffolds, vascular and microfluidic networks, chemical or physical regulation systems, bioelectronic interfaces, and feedback and control architectures. For each system, the control strategy is defined around the dynamics and biological operating window of the selected living component, while responsiveness and operational stability are validated over the intended functional lifetime.

The final stage of the BinD paradigm, comprising integration and prefabrication, merges the biological and synthetic elements into a unified system design. Crucially, it addresses spatiotemporal synchronization challenges to ensure that manufacturing processes, such as synthetic material curing, are compatible with biological viability. Interactions between subsystems are validated and optimized through modeling, simulation, and iterative testing, and the design is prepared for fabrication using processes capable of handling biological and synthetic materials in tandem.

The order of stages in the BinD paradigm is not strictly linear. Feedback from later stages can inform revisions that are needed in earlier stages, creating iterative loops that refine system design as its biological and synthetic requirements evolve. This process also enables the sustainability of the system to be reassessed and improved as the design develops. This flexibility is essential to ensure that the final system is adaptive and responsive to the inherent variability of living materials.

## 3 Implications of BinD across Domains

Most current workflows separate biological and synthetic components into sequential manufacturing domains, thereby limiting the ability to harness living dynamics as functional elements of the system [21]. In BinD biohybrids, design parameters and decisions evolve over time, as living systems continue to develop after deployment, effectively extending the fabrication process across their functional lifetimes. The BinD paradigm thus envisions unified fabrication ecosystems that coordinate bioprinting, additive manufacturing, microfluidic patterning, and self‐directed bioassembly within a single workflow. These ecosystems orchestrate the initial deposition of synthetic materials and the placement of biological matter to enable design‐informed growth trajectories, including delayed growth, degradation, and regenerative processes, thereby spawning capabilities that are unachievable with conventional sequential methods.

BinD enables machines that adjust their morphology and behavior in direct response to environmental signals, harnessing the intrinsic sensing, self‐assembly, and self‐healing mechanisms of living matter. Morphological and behavioral changes of this kind occur through biological reconfiguration and growth processes embedded within the system, rendering the BinD paradigm applicable across numerous high‐demand areas. For instance, similar principles support implants and therapeutic devices, critically enabling them to integrate with host tissues over time and to undergo continuous reconfiguration, opening broad opportunities for biointegratively designed systems across various healthcare applications. In addition, BinD‐biohybrids are excellent candidates for deployment in natural environments, where current robotic systems rely on synthetic materials that persist long after their functional lifetimes and thus accumulate across ecosystems. Biodegradable and living components constitute an alternative path that mirrors natural cycles, such that systems can be designed to decompose, regenerate, or be reabsorbed. Early visions of zero‐footprint eco‐robotics [2] and biodegradable sensing systems for ecological monitoring [22] were already headed conceptually in this direction. As autonomous systems expand across natural environments [23], the material life cycle of these machines becomes inseparable from their function.

As a prospective example, BinD could extend the previously proposed framework for autonomous ecological monitoring using biodegradable sensors [22] to a seed‐based plant biohybrid system for monitoring drought stress. During bioconceptualization, the system would be conceived for drone deployment into locations that are difficult to access, and a seed suited to the target environment would be selected as the living component, with growth beginning only after deployment and the required operating period and end‐of‐life pathway defined from the outset. Living‐subsystem design would characterize the selected seed and its growth dynamics, establish the conditions needed to preserve viability and initiate growth, and determine how the plant response used for sensing changes as the living component develops. The life‐supporting subsystem would preserve seed viability during fabrication, storage and deployment and then provide the conditions needed to initiate growth and sustain the living component throughout operation. The function‐supporting subsystem would enable deployment by drone, convert the plant response into a measurable signal, and transmit the resulting data. Integration and prefabrication would bring the dormant seed and biodegradable supporting components into a unified design that remains compatible with fabrication and allows growth to begin after deployment. From living‐subsystem design onward, the outcome of each stage would be validated and used to refine earlier choices until the system can sense reliably for the required period and its artificial components can safely degrade after use.

This example illustrates how BinD reframes the design of autonomous biohybrid systems. Existing approaches in systems engineering and biofabrication address parts of the problem, but they operate in different stages. The broadly known bioinspiration design paradigm follows an inspire‐abstract‐implement sequence in which biological principles guide the development of synthetic systems [12]. Whereas bioinspiration draws on biology as a source of principles, BinD integrates living matter itself as an active engineering component, in the frame of which growth, degradation, and adaptation are controllable design features.

Indeed, the BinD paradigm supports a form of circular engineering grounded in biological renewal. Material flows can be aligned with ecological processes such that the persistent accumulation of synthetic materials can be reduced by incorporating growth and decay cycles in biohybrid systems. BinD defines a framework for the construction of systems whose physical intelligence emerges from these dynamics and whose presence is compatible with the environments they inhabit.

At the same time, the adoption of BinD and the transition from conventional synthetic devices to sustainable biohybrid systems will transform product characteristics, necessitate consideration of their living dynamics, and consequently reshape business models, including those for manufacturing, commercialization, and end‐of‐life management. Today's industries design and build products that age, fail, and must eventually be replaced. BinD systems, in contrast, fundamentally challenge this paradigm, as they are designed to grow during and after deployment, self‐regenerate on failure, and degrade or resorb at the end of their functional lives. A robotic system that partially self‐assembles post‐deployment requires a different manufacturing scope. An implantable device that resorbs, for example, eliminates the need for revision surgeries and for associated device sales.

The BinD paradigm thus fosters conditions in which existing business models gradually become misaligned with the technology's life cycle, compelling manufacturers, healthcare providers, and regulators to develop new frameworks for value creation, liability, and end‐of‐life accountability. While today's products are typically discarded by users at the end of product life, biohybrid systems will likely require regulators to enforce mandatory biodisposal rules to ensure biosafety and proper biodegradation, potentially incurring additional costs for manufacturers or end users. Similar considerations are necessarily relevant to all applied biohybrid systems, dictating the need to rethink their respective business models.

## 4 Enabling the BinD Paradigm in Interdisciplinary Research

The limitations of current, disciplinarily isolated frameworks are highlighted by the cross‐domain shifts required by the BinD paradigm, which necessitate systemic changes across all impacted domains. The successful implementation of the BinD paradigm will require structural changes in how interdisciplinary research is supported and organized today. Current research systems remain strongly disciplinary, with funding schemes, hiring practices, and evaluation criteria typically aligned with narrow fields of expertise. As a result, work that integrates biology, engineering, and design at a fundamental level often faces structural barriers, including difficulties in aligning timelines, methodologies, and expectations across domains. As an early‐stage paradigm, BinD's broader adoption will require the structural and institutional developments outlined here. The structural landscape of the BinD paradigm (Figure 2) provides a scalable blueprint for the emergence of interdisciplinary fields, despite the constraints of institutional and communication silos.

**FIGURE 2:**
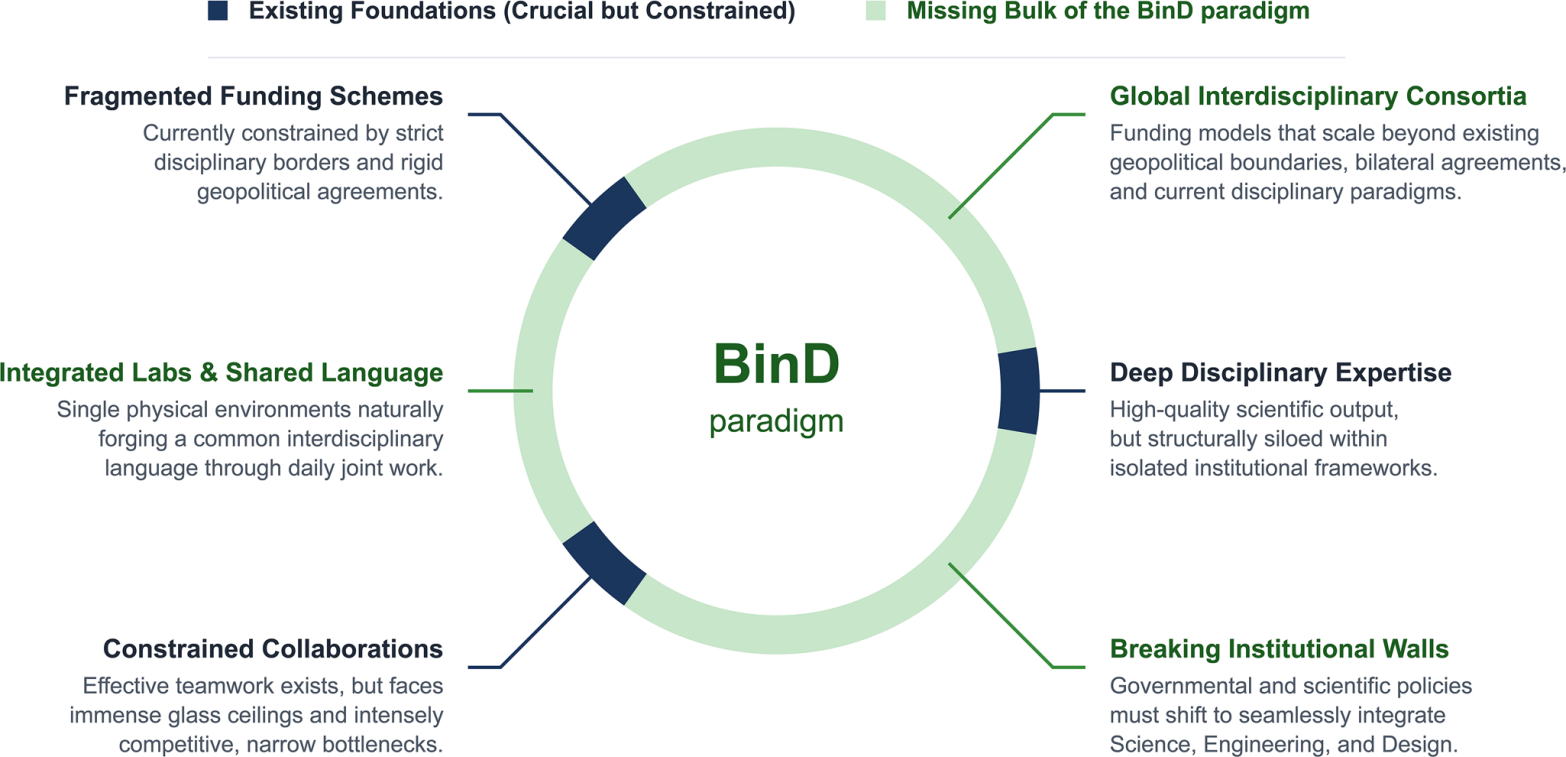
Structural landscape of the BinD paradigm and its scalability. BinD integrates the existing disciplinary foundations (blue) with the essential interdisciplinary components (green) currently absent from traditional frameworks. While presented in the context of Biointegrative Design, this structural model is a universal blueprint for other emerging interdisciplinary paradigms that dictate the need to dismantle institutional silos and to create shared cross‐domain languages.

Large, interdisciplinary consortia play an important role in connecting expertise across institutions and countries, particularly in areas that extend beyond laboratory integration, including the humanities, ethics, and law. Currently, however, such interdisciplinary initiatives are limited in number, and they are often shaped by geopolitical priorities, bilateral agreements, and existing collaboration networks. While these interdisciplinary (and often international) collaborations may constitute highly fertile ground for new ideas, the funding mechanisms to sustain them are not widely available, effectively stifling many promising lines of research. Such collaborations are often short‐lived and cannot survive in current budget‐driven research realities, given the severe lack of institutional schemes designed to sustain this type of work. Expanding and democratizing funding frameworks that enable and stabilize such interdisciplinary consortia will be essential to the implementation of BinD and other interdisciplinary approaches with which to achieve ambitious, crucial goals of humanity, such as sustainable life.

Beyond its technical efficiency, the iterative nature of the BinD paradigm supports effective regulatory and ethical oversight. Unlike traditional linear workflows, where compliance is often assessed only in the final system, BinD enables real‐time monitoring of biological components and their interactions across its four design stages. This setup ensures that potential ethical or biosafety risks are identified and addressed early in each iteration, which significantly streamlines the path to legal certification and social integration.

In parallel, supporting BinD requires mechanisms that enable integration at the laboratory level, where multiple disciplines can be developed and combined in a single research environment. Similar approaches have been explored in the context of Physical Artificial Intelligence, where materials science, mechanical engineering, computer science, biology, and chemistry are united in tightly integrated laboratory settings to enable the co‐development of materials, structures, and control systems [19]. Such environments facilitate rapid iteration and continuous cross‐disciplinary interaction.

For BinD, the development of shared, interdisciplinary languages plays a central role. Emerging through sustained collaboration between researchers from different fields working toward a common goal, these languages allow concepts, constraints, and design principles to migrate across disciplinary boundaries in ways that are difficult to formalize in advance. In this sense, interdisciplinary language formation occurs alongside BinD as one of its defining outcomes, shaping not only how problems are defined, communicated, and solved, but also how the designed system can be described as a whole. BinD thus addresses a fundamental gap in how living systems are conceived, built, and integrated into the world.

## Conflicts of Interest

The author declare no conflicts of interest.

## Data Availability

Data sharing not applicable to this article as no datasets were generated or analyzed during the current study.
